# Ascorbate-mediated regulation of growth, photoprotection, and photoinhibition in *Arabidopsis thaliana*

**DOI:** 10.1093/jxb/ery170

**Published:** 2018-05-03

**Authors:** William Plumb, Alexandra J Townsend, Brwa Rasool, Sarah Alomrani, Nurhayati Razak, Barbara Karpinska, Alexander V Ruban, Christine H Foyer

**Affiliations:** 1 Centre for Plant Sciences, School of Biology, Faculty of Biological Sciences, University of Leeds, Leeds, UK; 2 Department of Cell and Molecular Biology, School of Biological and Chemical Sciences, Queen Mary University of London, London, UK; 3 Technical College of Applied Science, Sulaimani Polytechnic University, Sulaimani, Kurdistan, Iraq

**Keywords:** Anthocyanin, antioxidants, NPQ, oxidation, photoinhibition, photosynthesis, ro-GFP, zeaxanthin

## Abstract

The requirements for ascorbate for growth and photosynthesis were assessed under low (LL; 250 µmol m^–2^ s^–1^) or high (HL; 1600 µmol m^–2^ s^–1^) irradiance in wild-type *Arabidopsis thaliana* and two ascorbate synthesis mutants (*vtc2-1* and *vtc2-4*) that have 30% wild-type ascorbate levels. The low ascorbate mutants had the same numbers of leaves but lower rosette area and biomass than the wild type under LL. Wild-type plants experiencing HL had higher leaf ascorbate, anthocyanin, and xanthophyll pigments than under LL. In contrast, leaf ascorbate levels were not increased under HL in the mutant lines. While the degree of oxidation measured using an *in vivo* redox reporter in the nuclei and cytosol of the leaf epidermal and stomatal cells was similar under both irradiances in all lines, anthocyanin levels were significantly lower in the low ascorbate mutants than in the wild type under HL. Differences in the photosynthetic responses of *vtc2-1* and *vtc2-4* mutants were observed. Unlike *vtc2-1*, the *vtc2-4* mutants had wild-type zeaxanthin contents. While both low ascorbate mutants had lower levels of non-photochemical quenching of chlorophyll *a* fluorescence (NPQ) than the wild type under HL, qP_d_ values were greater only in *vtc2-1* leaves. Ascorbate is therefore essential for growth but not for photoprotection.

## Teaser

Analysis of synthesis mutants demonstrates an ascorbate requirement for growth under low light and for high light-dependent anthocyanin accumulation, but no consistent effects on photoinhibition or zeaxanthin accumulation were found.

## Introduction

The low molecular weight antioxidant ascorbate (vitamin C) is considered to fulfil a number of important functions in plants (Smirnoff, 2000; Foyer and Noctor, 2000, 2009; Gallie, 2013). Although different pathways of ascorbate synthesis have been described in higher plants, the l-galactose pathway (also known as the Wheeler–Smirnoff pathway) is the major pathway operating in Arabidopsis leaves (Wheeler *et al.*, 1998). The rate-limiting step in this pathway is the conversion of GDP-l-galactose to l-galactose-1-phosphate which is catalysed by the enzyme GDP-l-galactose phosphorylase. This enzyme, which catalyses the first ascorbate-specific step in the pathway, is encoded by two paralogous genes, *VTC2* and *VTC5.* The pathway of ascorbate synthesis is regulated by light (Yabuta *et al.*, 2007). Sun-exposed leaves accumulate substantially more ascorbate than shade leaves (Smirnoff and Pallanca, 1996). Moreover, leaf ascorbate accumulation shows diurnal variations (Dutilleul *et al.*, 2003; Pignocchi *et al.*, 2003; Tamaoki *et al.*, 2003; Tedone *et al.*, 2004), with the quantity and quality of light experienced during the photoperiod modulating the extent of ascorbate accumulation (Bartoli *et al.*, 2009). Relatively few factors that are involved in the regulation of ascorbate synthesis have been identified to date. Of these, ascorbic acid mannose pathway regulator (AMR) 1 regulates the expression of genes involved in ascorbate synthesis in response to developmental and environmental cues (Zhang *et al.*, 2009). Ethylene response factor (ERF) 98 is a positive regulator of ascorbate synthesis pathway genes (Zhang *et al.*, 2012). Transgenic plants overexpressing *AtERF98* showed enhanced expression of ascorbate synthesis genes, while the knockout *aterf98-1* mutants showed a lower capacity for ascorbate synthesis in the absence or presence of salt stress (Zhang *et al.*, 2012). The COP9 signalosome (CSN) is a photomorphogenic complex that functions in the ubiquitin–proteasome pathway through regulation of E3 ligase activity, and is involved in the degradation of ascorbate biosynthetic enzymes in the dark (Wang *et al.*, 2013).

Ascorbate fulfils a number of diverse roles in the regulation of photosynthesis, particularly in the acclimation of plants to high light (Müller-Moulé *et al.*, 2003, 2014; Karpinska *et al.*, 2017*a*). First, ascorbate is an important component in the water–water cycle, which protects against photoinhibition. In this process, the Mehler reaction provides an alternative electron sink generating superoxide anion radicals, O_2_·^−^, that are dismutated to hydrogen peroxide by the action of the thylakoid copper/zinc superoxide dismutase. Hydrogen peroxide is then reduced to water by ascorbate peroxidases (APXs) and the chloroplast 2-Cys peroxiredoxins (PRXs; Awad *et al.*, 2015). The water–water cycle not only scavenges superoxide and hydrogen peroxide, but it also functions to dissipate excess excitation energy and electrons (Foyer *et al.*, 1991; Asada, 2000). The importance of the water–water cycle as an electron sink (Osmond and Grace, 1995) should be viewed, however, in terms of its contribution to thylakoid acidification and the control of PSII activity (Heber, 2002). Secondly, ascorbate is a potent specific inhibitor of *2CPA* expression (Horling *et al.*, 2003; Baier *et al.*, 2004), influencing chloroplast to nucleus signalling pathways via the redox-sensitive transcription factor Rap2.4a (Shaikhali *et al.*, 2008). Thirdly, ascorbate is able to donate as well as accept electrons from the photosynthetic electron transport chain acting as an alternative electron donor for PSII (Mano *et al.*, 2004; Tóth *et al.*, 2009).

Fourthly, ascorbate is required for the regeneration of lipid-soluble antioxidants, particularly the tocopherols and tocotrienols (vitamin E), which protect the polyunsaturated fatty acids in the thylakoid membranes from singlet oxygen. During oxidation, tocopherols become oxidized to chromanoxyl radicals, which can be converted back to vitamin E by the reducing power of ascorbate or by reaction with carotenoids. Finally, ascorbate is required for the conversion of violaxanthin to zeaxanthin in the light-dependent xanthophyll cycle, which participates in the thermal dissipation of energy under high light (Bratt *et al.*, 1995; Müller-Moulé *et al.*, 2003; Jahns *et al.*, 2009). Knockout mutants of the chloroplast envelope ascorbate transporter AtPHT4;4 were shown to be compromised in thermal energy dissipation (Miyaji *et al.*, 2015).

The *vtc2-1* mutant carries a single base substitution (G to A) in the predicted 3' splice site of the fifth intron of the *VTC2* gene, resulting in a 90% reduction in transcript levels compared with the wild type (Dowdle *et al.*, 2007). Previous studies have shown that in *Arabidopsis thaliana*, the *vtc2-1* mutants grow well under HL but acclimation of photosynthesis is impaired compared with the wild-type plants in terms of *F*_v_/*F*_m_, *P*_max_, and ϕPSII (Müller-Moulé *et al.*, 2014). While such findings confirm that ascorbate has a protective effect on the photosynthetic processes, the precise mechanisms involved remain poorly characterized. Similarly, only one mutation in the *VTC2* gene (*vtc2-1*) has previously been characterized in relation to the regulation of photosynthesis, and a degree of caution must be exercised in the interpretation of data arising from the analysis of single mutants (Lim *et al.*, 2016). The following experiments were therefore conducted to determine the influence of low ascorbate on photoprotection and photoinhibition in two ascorbate synthesis mutants (*vtc2-1* and *vtc2-4*) with defects in the *VTC2* gene. The *vtc2-1* and *vtc2-4* mutants have a different shoot growth phenotype when grown under continuous light conditions although they have the same low level of leaf ascorbate compared with the wild type (de Simone *et al.*, 2015; Lim *et al.*, 2016). Unlike *vtc2-1*, *vtc2-4* is a T-DNA insertion mutant with a complete loss of function. However, the *VTC5* gene still provides sufficient residual GDP-l-galactose phosphorylase activity in the *vtc2-4* mutants to allow a reduced level of ascorbate synthesis and accumulation.

Light-induced decreases in photosynthetic capacity are often reflected by permanent damage (photodamage) and resultant closure of PSII reaction centres (RCIIs) (Powles, 1984). Assessment of the different components and pathways contributing to the photoprotection of the photosynthetic membrane has been difficult due to the lack of consensus of the molecular mechanisms involved, the presence of multiple pathways occurring over different time scales, and the difficulty in accurately measuring and quantifying them (Aro *et al.*, 1993; Jahns and Holzwarth, 2012; Ruban and Murchie 2012; Tyystjärvi, 2013). For example, measurements have previously focused on disruptive light treatments or invasive techniques (i.e. western blots) in order to estimate and evaluate photodamage (Greenberg *et al.*, 1987; Tyystjärvi and Aro, 1996). An improved, relatively rapid, and non-destructive protocol was therefore developed to measure photochemical quenching of chlorophyll *a* fluorescence (NPQ), which can provide information of the effectiveness of protective processes in the photosynthetic membrane (Ruban and Murchie, 2012). The method involves using pulse amplitude modulation (PAM) fluorometry to impose a gradually increasing actinic light routine to track the yield of chlorophyll fluorescence and infer the onset of photoinactivation (often leading to photodamage) *in vivo* (see the Materials and methods). This enables the evaluation of the role of ascorbate in providing photoprotective effectiveness to the photosynthetic processes in the thylakoid membranes. The data presented here demonstrate that the low leaf ascorbate in the *vtc2-1* and *vtc2-4* mutants led to a slow growth phenotype in plants grown under short-day (8 h day/16 h night) low-light (LL; 250 µmol m^–2^) conditions. Similarly, anthocyanin accumulation was restricted by low ascorbate in both mutant lines under high-light (HL) growth conditions. Only the *vtc2-1* mutant and not the *vtc2-4* mutants accumulated less zeaxanthin under HL. Moreover, the level of HL-induced photodamage was not significantly higher in either of the low ascorbate mutants compared with the wild type.

## Materials and methods

### Plant material and growth conditions

Seeds of *A. thaliana* ([L.] Heynh.) wild-type ecotype Columbia-0 (Col-0) and *vtc2-4* (SAIL_769_H05) (Lim *et al.*, 2016) were obtained from the Nottingham Arabidopsis Stock Centre (NASC). Seeds of the *vtc2-1* mutant (Conklin *et al.*, 2000) were obtained from Dr Robert Last (USA). Seeds of transgenic *A. thaliana* (L.) that constitutively express redox-sensitive green fluorescent protein2 (roGFP2) and *vtc2-1*roGFP2 were as described in de Simone *et al.* (2017)

Unless otherwise stated, plants were grown in compost in controlled-environment chambers at constant relative humidity (60%) and temperature (20 ^o^C) under an 8 h day/16 h night. Plants were grown for the time specified in the figure legends under LL (250 µmol m^–2^ s^–1^), after which half of the plants were transferred to HL (1600 µmol m^–2^ s^–1^) for a further 7 d while the other half were maintained under LL conditions. Illumination in the controlled-environment chambers was provided by Attis-7 LED lights (Plessey, Plymouth, UK).

### Shoot phenotype

Ten independent plants per genotype and growth irradiance were measured at the end of the photoperiod at the times indicated in the figure legends. Rosette diameters were measured with a ruler and the numbers of leaves per rosette were counted. Leaf area was calculated from digital images of the rosettes. In younger rosettes, this measurement accurately measures leaf area but it is less accurate in older rosettes due to some overlap of leaves. The rosettes were then photographed using a Canon EOS 450D digital camera mounted at a set height above the pots. The digital images captured were used to estimate the number of fully expanded leaves, and rosette areas were estimated using the Image J program version 1.41a scaled to a ruler placed alongside each image.

### Pigment analysis

Photosynthetic pigments were estimated in the youngest fully expanded leaves harvested from three independent 4-week-old plants per genotype per time point. Leaves were weighed and ground in liquid nitrogen, and ice-cold 95% ethanol was added at a ratio of 10 ml g^–1^ leaf FW. Extracts were centrifuged for 10 min at 14 000 *g* (4 °C) and the supernatant fractions were used for pigment determination. Chlorophyll and carotenoids were measured by spectrophotometry according to the method of Lichtenthaler (1986). Xanthophyll pool sizes and the de-epoxidation state (zeaxanthin+0.5×antheraxanthin)/(violaxanthin+antheraxanthin+zeaxanthin) were determined by reversed-phase HPLC using a LiChrospher 100 RP-18 column (Merck) and a Dionex Summit chromatography system as previously described (Ruban *et al.*, 1998). Anthocyanins were extracted and assayed as described by Neff and Chory (1998).

### Whole-leaf ascorbate and dehydroascorbate

Whole-leaf samples from three independent plants per genotype per time point (as indicated in the figures) were harvested, weighed, and immediately ground in liquid nitrogen under the prevailing dark/light conditions in the controlled-environment chambers. Ascorbate and dehydroascorbate (DHA) were extracted from the frozen pellets by grinding again in 1 M HClO_4_ at a ratio of 10 ml g^–1^ FW and assayed as described by Queval and Noctor (2007). Leaf ascorbate and DHA were calculated as described by Noctor *et al.* (2016).

### Fluorescence measurements

The protective effectiveness of processes occurring in the photosynthetic membrane was assessed using the NPQ method (Ruban and Murchie, 2012), which entails a gradually increasing actinic light routine to track the relationship between yield (ϕ_PSII_), NPQ, and qP_d_ (the quantum coefficient of photochemical quenching measured in the dark following light exposure (Fig. 6). ϕ_PSII_ was undermined by NPQ and the photoinactivation of RCII according to Equation 1.

$$\phi PSII=\frac{qPd.\frac{Fv}{Fm}}{\left[1+\left(1-\frac{Fv}{Fm}\right).NPQ\right]}$$ 1

where *F*_v_/*F*_m_ is the maximum photochemical quantum yield of PSII, calculated as [*F*_m_–*F*_o_]/*F*_m_, and *F*_m_ and *F*_o_ are the maximum and minimum yields of fluorescence, respectively. NPQ is calculated as (*F*_m_–*F*_m_')–1. The qP_d_ parameter is a measure of the coefficient of photochemistry in the dark, is calculated according to Equation 2:

$$qPd=\frac{\left(Fm^{\text{'}}-Fo^{\text{'}}{}_{act}\right)}{\left(Fm^{\text{'}}-Fo^{\text{'}}{}_{calc}\right)}$$ 2

where *F*_o_'_act_ and *F*_o_'_calc_ are the actual and calculated minimum levels of fluorescence in the dark following actinic light (AL) illumination. *F*_o_'_calc_ is quantified according to Equation 3:

$$Fo^{\text{'}}{}_{calc}=\frac{1}{\frac{1}{Fo}-\frac{1}{Fm}\;+\frac{1}{Fm\text{'}}}$$ 3

### Procedures

pNPQ (protective NPQ) measurements on whole intact leaves were performed using a JUNIOR-PAM fluorimeter (Walz, Effeltrich, Germany) and a magnetic leaf clip. Plants were dark adapted for 45 min before each procedure. The procedure used AL intensities of 150, 317, 475, 700, 1042, 1367, 1917, and 2500 µmol photons m^–2^ s^–1^. Each AL increment lasts for 5 min, before a saturating pulse (SP) is applied in the light to measure the NPQ. The AL is then turned off for 10 s but with far-red (FR) light on, before another SP is applied to measure the photochemical quenching in the dark (qP_d_). After this SP, the AL is turned on for another 5 min at the next AL intensity. The procedure was run as a pre-programmed batch file with the scheme: (SP)-(AL on)-(120 s)-(SP)-(180 s)-(SP)-(AL off/FR on)-(7 s)-(SP)-(5 s)-(AL on/FR off)-repeat. Under low light intensities, the *F*_o_'_calc_ matches *F*_o_'_act_; however, under high light intensities, the two *F*_o_' values diverge. This is due to the rise in minimum fluorescence caused by the closure of RCII. This causes *F*_o_'_calc_<*F*_o_'_act_ and qP_d_<1.00, and at this point the leaf is considered to be photodamaged. A qP_d_ value of 1.00 represents 100% open reaction centres (RCs). However, to account for the natural variations in qP_d_, values <0.98 were selected as a mark of photodamage (i.e. >2% of RCIIs are damaged), with damage being relatively proportional to the decline in qP_d_ (Ware *et al.*, 2015). When qP_d_>0.98, NPQ is considered to be protective and is thus called pNPQ. For a detailed description of the principles of the method, see Ruban and Murchie (2012). The pNPQ measurements were taken before, during, and after the light treatment. Five replicates were taken per line at three different time points during the experiment: prior to light treatment, following 7d of light treatment, and 24 h after plants were returned to the lower light intensity, hereby referred to as before treatment, during treatment, and the recovery stage.

### roGFP measurements

Batches of roGFP2 and *vtc2-1*roGFP2 seedlings were grown for 7 d on vertical agar plates containing half-strength Murashige and Skoog medium with 0.1 g l^–1^ myoinositol, 10 g l^–1^ sucrose, and 0.5 g l^–1^ MES buffer (pH 5.7) as described previously (Kerchev *et al.*, 2011). Seedlings were grown either under LL (250 µmol m^–2^ s^–1^) for 7 d or under LL for 6 d followed by HL (1600 µmol m^–2^ s^–1^) for 24 h. For confocal microscopy, seedlings were placed on a slide in a drop of sterile water. Fluorescence imaging was performed using a Carl Zeiss confocal microscope (Carl Zeiss LSM880, Jena, Germany). The microscope was equipped with 405 nm and 488 nm lasers for detection of the oxidized and reduced forms of ro-GFP2, respectively. Images were taken with a ×40/1.3 Oil DIC M27 lens (Zeiss Objective C-Apochromat ×40/1.2 W Corr M27) in multitrack mode with line switching between 488 nm and 405 nm excitation. Ratiometric analyses were performed using ImageJ software (http://rsbweb.nih.gov/ij/). The range of the roGFP2 signal was calibrated at the end of each experiment using 2.5 mM DTT (reduced) or 2 mM hydrogen peroxide (oxidized). The oxidation degree and glutathione redox potential values were calculated as described in de Simone *et al.* (2017). Each experiment involved 50 seeds per line and was repeated three times.

### Statistics

ANOVA was carried out on all parameters at each growth stage using GenStat for Windows, 17th Edition (VSN International Ltd). Data were checked to see if they met the assumption of constant variance and normal distribution of residuals.

## Results

### Growth phenotypes of wild-type, *vtc2-1*, and *vtc2-4* plants grown under low light

**Fig. 1.**
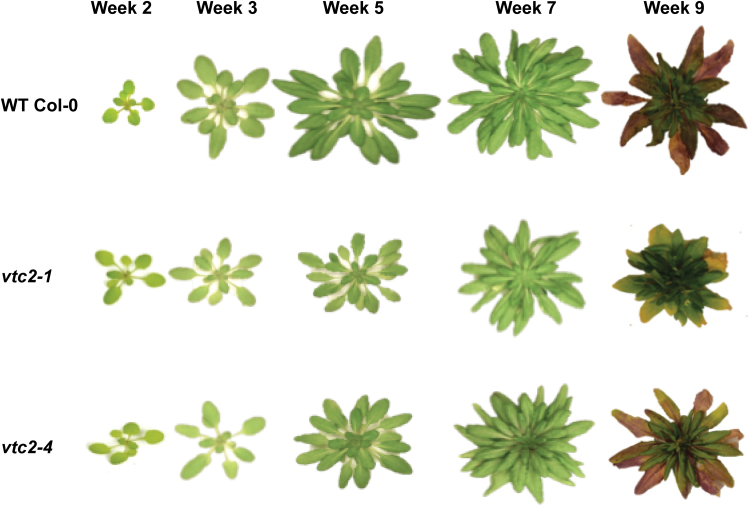
A comparison of the rosette phenotypes of wild-type (WT) *A. thaliana*, and the *vtc2-1* and *vtc2-4* mutants grown under low light (LL) conditions. Plants were grown for up to 9 weeks under LL (250 μmol m^–2^ s^–1^) conditions.

**Fig. 2.**
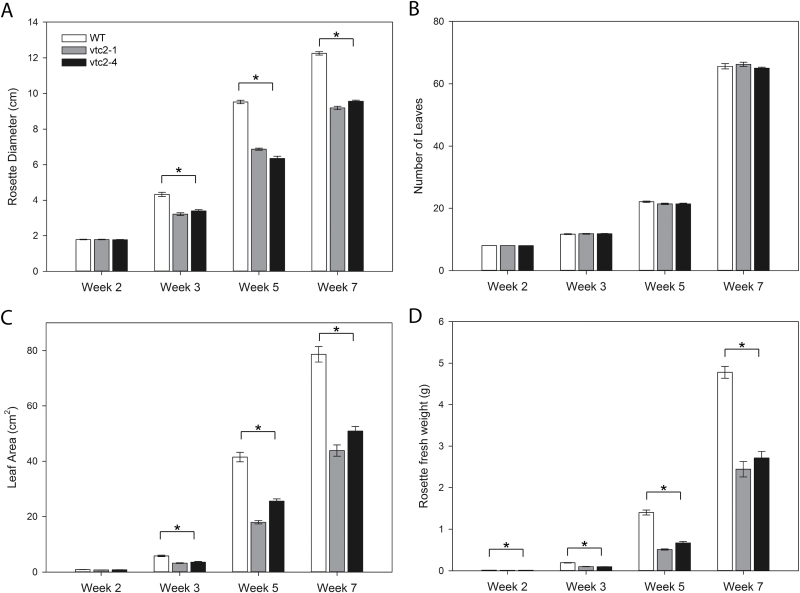
Rosette phenotypes of wild-type (WT), *vtc2-1,* and *vtc2-4 A. thaliana* genotypes grown under low light (250 μmol m^–2^ s^–1^) conditions for 7 weeks. (A) Rosette diameter. (B) Number of leaves. (C) Leaf (rosette) area. (D) Biomass (fresh weight). Error bars represent the SE (*n*=10). * indicates significantly different results according to ANOVA, *P*<0.05.

The rosettes of the *vtc2-1* and *vtc2-4* mutants were visibly smaller than those of the wild type when grown under LL (250 µmol m^–2^ s^–1^) conditions, particularly at the later stages of development (Fig. 1). Although the *vtc2-1* and *vtc2-4* mutants had a smaller rosette diameter (Fig. 2A), with a smaller total leaf area (Fig. 2C) and leaf biomass (Fig. 2C), they had the same number of leaves as the wild type at equivalent stages of development (Fig. 2B). Moreover, there were no visually detectable differences in rosette morphology (petiole length and leaf angle) between the lines.

### Leaf chlorophyll and total carotenoid contents in wild-type, *vtc2-1*, and *vtc2-4* plants

**Fig. 3.**
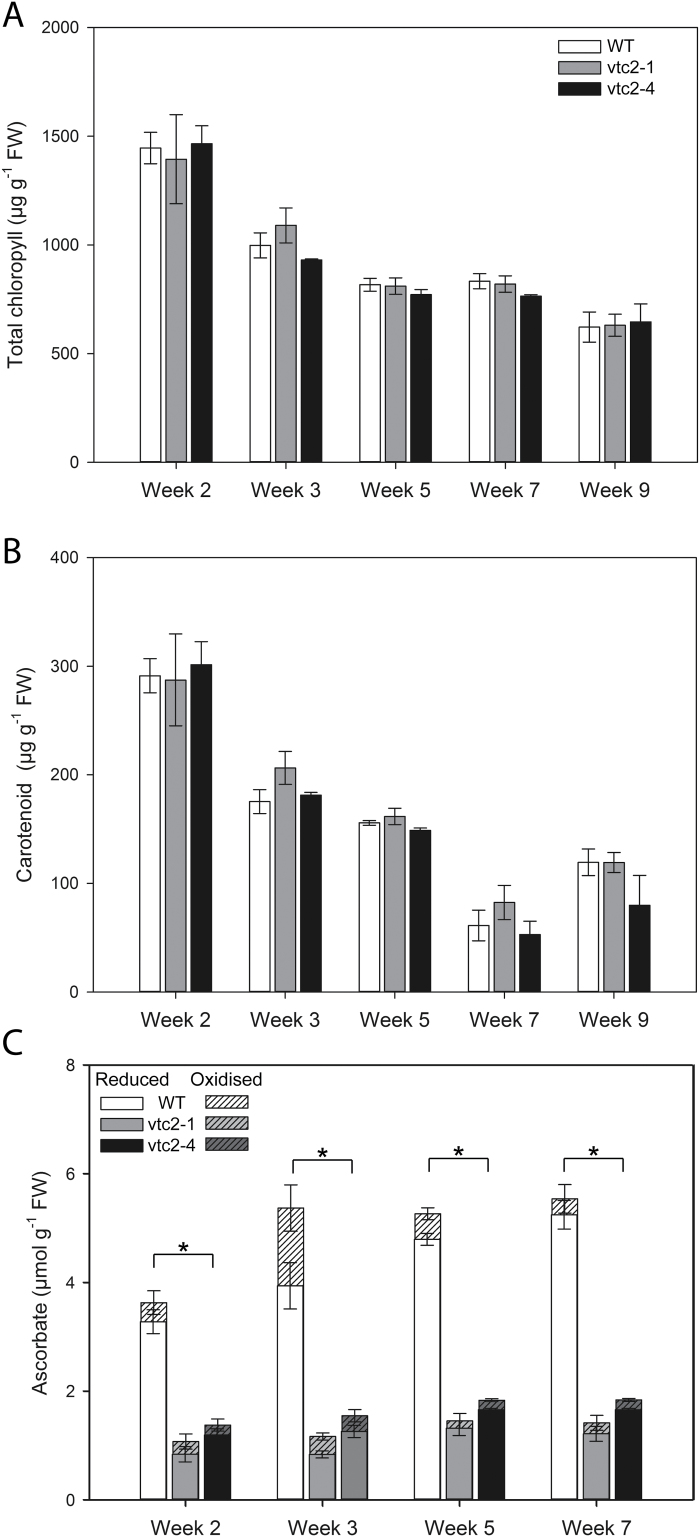
Developmental profiles of leaf pigments and ascorbate contents in wild-type (WT), *vtc2-1*, and *vtc2-4 A. thaliana* rosettes. Plants were grown under low light (250 μmol m^–2^ s^–1^) conditions for 9 weeks. (A) Chlorophyll. (B) Carotenoid pigments. (C) Ascorbate; reduced ascorbate (open bars), dehydroascorbate (striped bars). Error bars represent the SE (*n*=9). * indicates significantly different results according to ANOVA, *P*<0.05.

**Fig. 4.**
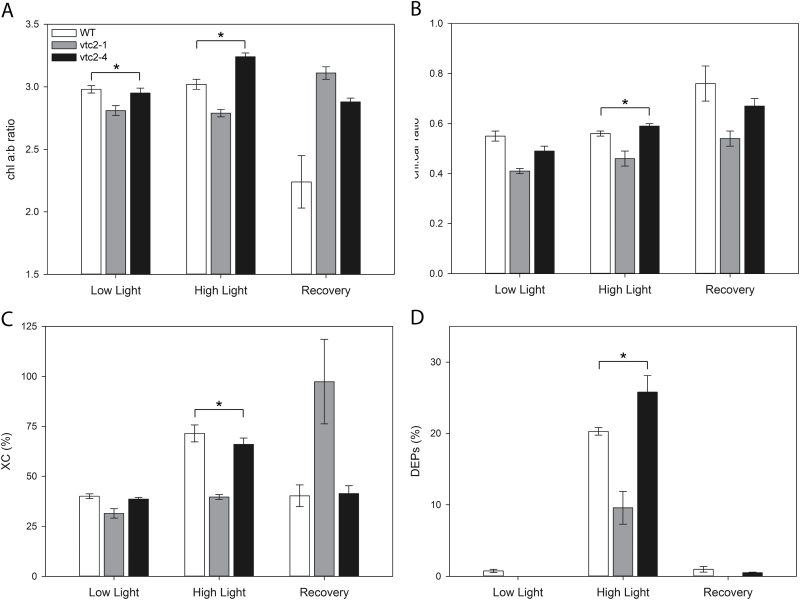
The pigment composition of the *vtc2-1* and *vtc2-4* leaves compared with the wild type (WT). Plants were grown under low light (LL; 250 μmol m^-2^ s^-1^) conditions for 5 weeks and then either for a further 7 d under LL conditions or after transfer to high light (HL; 1600 μmol m^–2^ s^–1^) growth conditions for 7 d. Leaves were harvested from plants grown only under LL, after 7 d growth under HL, and after ‘recovery’ (2 d after return to the LL growth environment after 7 d under HL). De-epoxidation state (DEPs)=(Z+0.5A)/(V+A+Z); XC: xanthophyll pool (Z+A+V)/(Z+A+V+N+L+β-car) where Z, zeaxanthin; V, violaxanthin; A, antheraxanthin; N, neoxanthin; L, lutein; β-car, β-carotene. Data are presented as means ± SEM from five replicates. * indicates significantly different results during each experimental time point according to ANOVA, *P*<0.05.

The *vtc2-1* and *vtc2-4* mutants had similar amounts of leaf chlorophyll (Fig. 3A) and carotenoid pigments (Fig. 3B) to the wild type when grown under LL conditions. The *vtc2-4* and *vtc2-1* leaves has similar levels of leaf ascorbate, the ascorbate levels of both mutants being significantly lower than that of the wild type at all stages of leaf development (Figs 3C, 4). The ratio of reduced to oxidized ascorbate was similar in all genotypes under LL growth conditions at all the stages of rosette development measured (Fig. 3C).

### The effect of low ascorbate on HL-dependent changes in leaf carotenoid pigment composition

The low ascorbate mutants showed differences in pigment composition compared with the wild-type (Fig. 4). However, there were few consistent differences between the *vtc2-1* and *vtc2-4* leaves relative to the wild type. Both low ascorbate mutants had significantly higher Chl *a*/*b* ratios (Fig. 4A) and lower chlorophyll to carotenoid ratios (Fig. 4B) than the wild type in the recovery phase at LL after growth under HL conditions. The xanthophyll pool size was lower in the *vtc2-1* mutant than in the wild type but higher in *vtc2-4* under HL and in the recovery phase at LL after growth under HL conditions (Fig. 4C). Moreover, the *vtc2-1* leaves had lower zeaxanthin levels, expressed as the de-epoxidation state of the xanthophyll pool, than the wild type or *vtc2-4* mutants under HL (Fig. 4D). Leaf zeaxanthin contents expressed as a percentage of the total carotenoid were greatly increased in the wild type and *vtc2-4* mutants under HL compared with LL or in the recovery period (Fig. 4D). In contrast, the *vtc2-1* leaves accumulated less zeaxanthin than the wild type or the *vtc2-4* mutant under HL (Fig. 4D).

### Effects of high light on leaf ascorbate levels

**Fig. 5.**
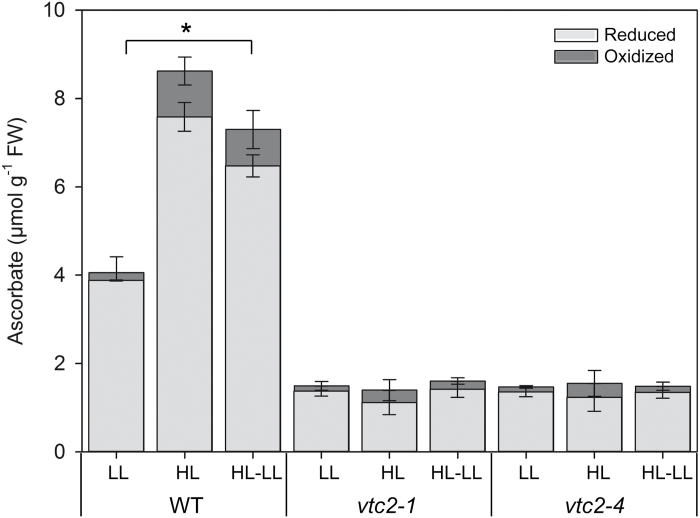
The effect of 7 d growth under high light (HL; 1600 µmol m^–2^ s^–1^) on the ascorbate levels in the leaves of the *vtc2-1* and *vtc2-4* mutants compared with the wild type. Plants were grown under low light (LL; 250 μmol m^–2^ s^–1^) conditions for 5 weeks and then batches of plants were either grown for a further 7 d under LL conditions prior to measurement or transferred to HL growth conditions for a further 7 d. Thereafter, some batches of HL-grown plants were returned to LL growth conditions for 2 d prior to measurement of reduced ascorbate and dehydroascorbate. Error bars represent the SE (*n*=9). * indicates significantly different results according to ANOVA, *P*<0.05.

**Fig. 6.**
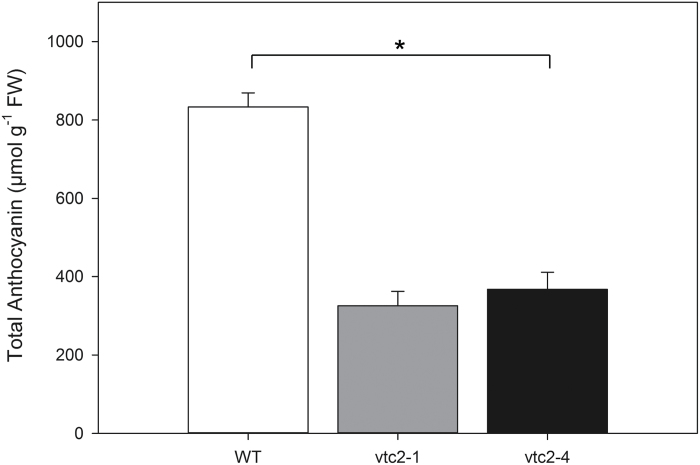
The effect of 7 d growth high light (HL; 1600 µmol m^–2^ s^–1^) on the levels of anthocyanin pigments in the leaves of the *vtc2-1* and *vtc2-4* mutants compared with the wild type. Plants were grown under low light (LL; 250 μmol m^–2^ s^–1^) conditions for 5 weeks and then either grown for a further 7 d under LL conditions prior to measurement or transferred to HL growth conditions for a further 7 d. * indicates significantly different results according to ANOVA, *P*<0.05. Error bars represent the SE (*n*=9).

The levels of ascorbate in the leaves of the wild-type plants were significantly increased by growth under HL compared with LL conditions (Fig. 5). The level of DHA also increased under HL compared with LL conditions. The levels of leaf ascorbate in the wild-type plants were decreased 48 h after return to the LL conditions. In contrast to the wild type, the leaves of the *vtc2-1* and *vtc2-4* mutants were not changed by HL light growth conditions, remaining at the same levels as plants grown under LL (Fig. 5). The levels of anthocyanins were below the levels of detection in the leaves of all genotypes grown under LL conditions (data not shown). After 7 d of growth under HL, the levels of anthocyanin pigments were much lower in the *vtc2-1* and *vtc2-4* leaves than in those of the wild type (Fig. 6).

### Effects of low ascorbate on NPQ and photodamage in wild-type, *vtc2-1*, and *vtc2-4* plants

**Fig. 7.**
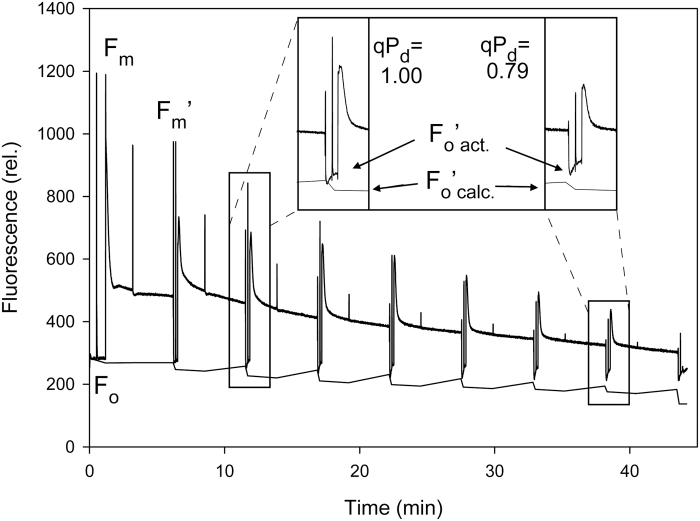
Scheme of induction of chlorophyll fluorescence from a WT Col-0 plant with an eight step actinic light (AL) routine. Inset: the gradually increasing AL routine induces photodamage which can be readily observed as a divergence between *F*_o_'_act_ and *F*_o_'_calc_, and be seen as a decrease in the qP_d_ parameter.

**Fig. 8.**
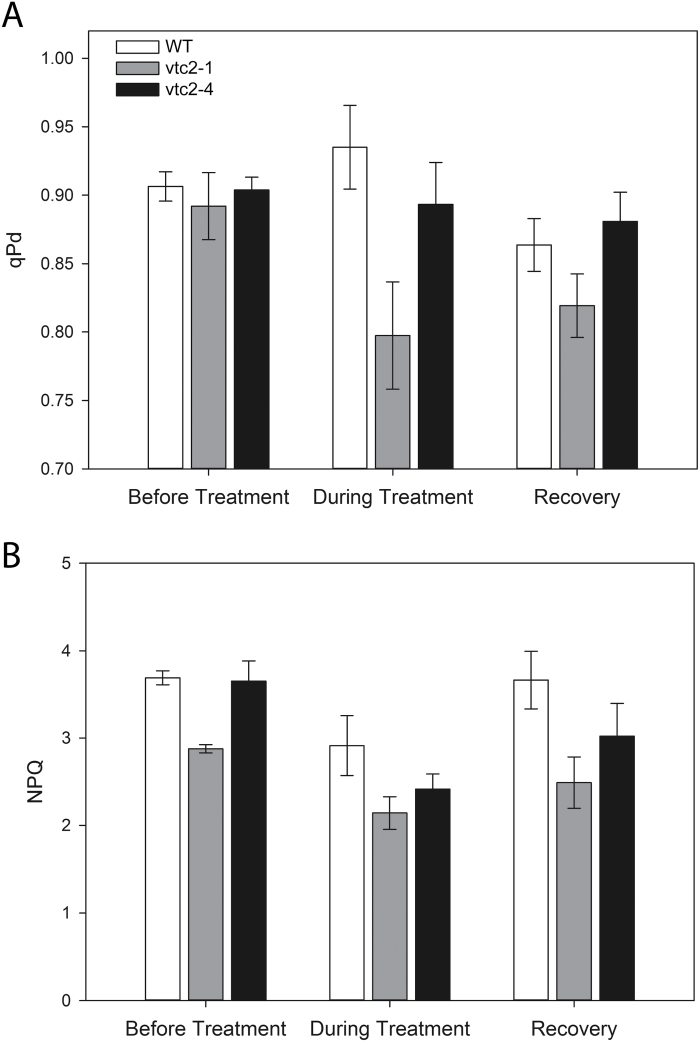
The photochemical quenching in the dark (qPd) and maximum NPQ values in the leaves of the *vtc2-1* and *vtc2-4* mutants compared with the wild type. Plants were grown under low light (LL; 250 μmol m^–2^ s^–1^) conditions for 5 weeks and then either grown for a further 7 d under LL conditions prior to measurement or transferred to high light (HL; 1600 μmol m^–2^ s^–1^) growth conditions for a further 7 d. A. The average qPd value at the end of the fluorescence routine presented in Fig. 6 taken before and during high light treatment and in the recovery phase. (B) The maximum NPQ value at the end of the fluorescence routine. Error bars represent the SE (*n*=5). * indicates significantly different results according to ANOVA, *P*<0.05.

Photodamage was quantified by the divergence in *F*_o_'_act_ and *F*_o_'_calc_, and the corresponding decline in the qP_d_ parameter, as illustrated in Fig. 7. A qP_d_ value of 1.00 represents 100% open RCs. Values <0.98 are considered to reflect photodamage (i.e. >2% of RCIIs are damaged). The loss of RC functions is proportional to the decline in qP_d_ (Ware *et al.*, 2015). The mutant and the wild-type leaves had similar qP_d_ values under LL and hence similar low levels of photodamage (Fig. 8A). Only the *vtc2-1* mutant showed a significant decrease in qP_d_ relative to the wild type (*P*<0.05) under the HL treatment. The data from both mutants have to be assessed together to determine the impact of ascorbate on photodamage. The *vtc2-4* plants had similar qP_d_ values to the wild type under HL and hence had similar levels of photodamage (Fig. 8A). During the recovery phase, whilst the qP_d_ appears to be lower in *vtc2-1* relative to the other lines, this difference is not significant.

The *vtc2-1* leaves exhibited significantly lower maximum NPQ values under LL than the wild type or the *vtc2-4* mutants under LL (Fig. 8B). The leaves of the wild type had higher NPQ values than those of the *vtc2-1* and *vtc2-4* mutants under HL conditions and during recovery from HL (Fig. 7B), maximum NPQ being generally lower under HL than LL growth conditions (Fig. 8B). During the recovery phase, the NPQ values were significantly higher in the wild type than in the *vtc2-1* and *vtc2-4* mutants.

### Effects of low ascorbate on the oxidation state of the nuclei and cytosol of stomatal and epidermal cells under LL and HL conditions

**Fig. 9.**
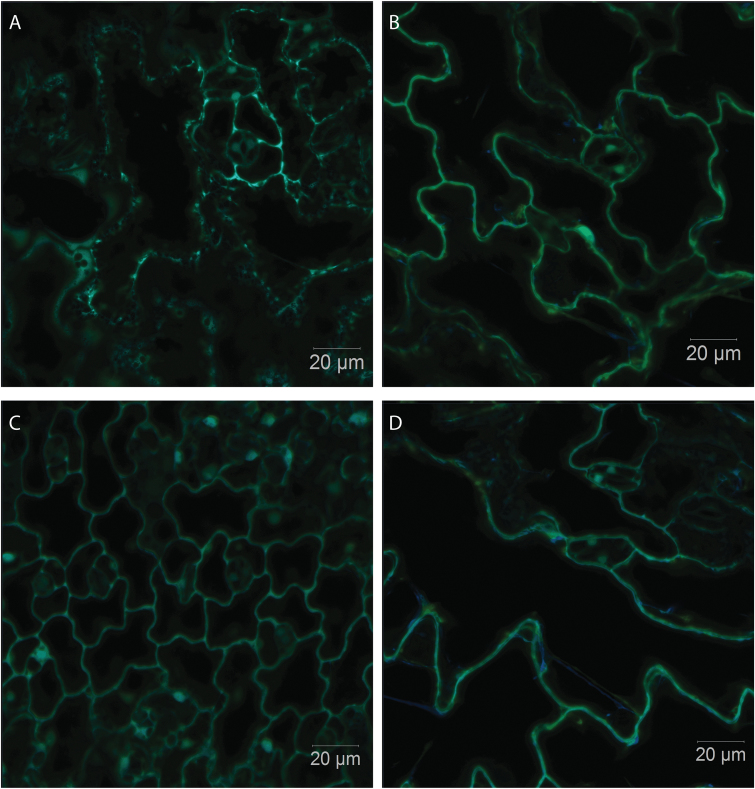
Overlaid images of the reduced and oxidized forms of ro-GFP2 fluorescence images of the cotyledons of *A. thaliana* wild-type seedlings expressing roGFP (A, C) and *vtc2-1* mutants expressing roGFP (*vtc2-1*roGFP; B, D). Seedlings had been grown on plates either under low light for 7 d (A, B) or under low light for 7 d followed by high light for 24 h (C, D). Scale bar=20 µm.

**Fig. 10.**
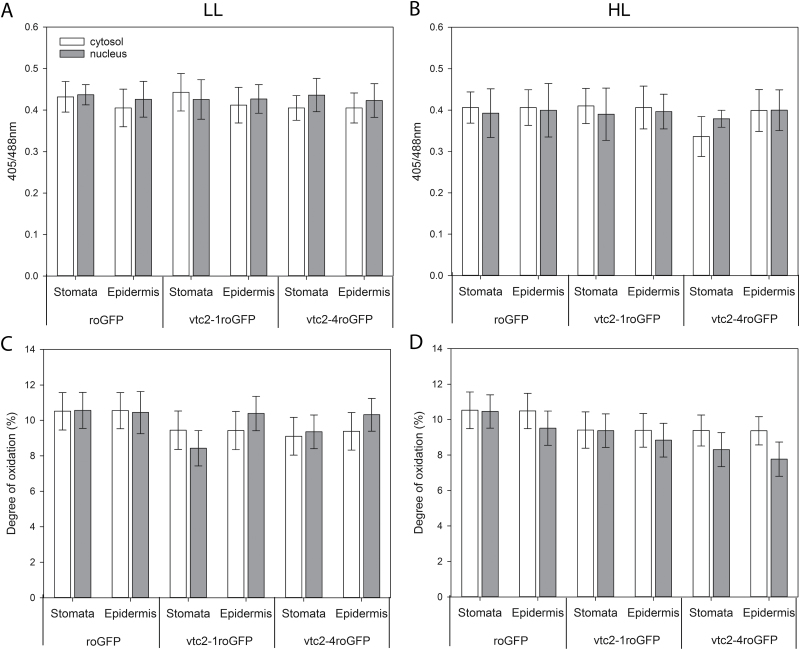
The 405/488 nm fluorescence ratios (A, B) and the degree of oxidation (C, D) measured in the cytosol (dark bar) and in the nuclei (grey bar) of the stomatal and epidermal cells of *A. thaliana* wild-type seedlings expressing roGFP, the *vtc2-1* mutants expressing roGFP (*vtc2-1*roGFP), and the *vtc2-4* mutants expressing (*vtc2-4*roGFP). Seedlings had been grown on plates either under low light for 7 d (A, C) or under low light for 7 d followed by high light for 24 h (B, D).

The in vivo redox state of the leaf cells was measured using roGFP (Fig. 9). The 405/488 nm fluorescence ratios (Fig. 10A, B), which provide a quantitative assessment in leaf epidermal and stomatal cells, were compared in wild-type seedlings expressing roGFP, the *vtc2-1* mutants expressing roGFP (*vtc2-1*roGFP), and *vtc2-4* mutants expressing roGFP (*vtc2-4*roGFP). The degree of oxidation was similar in the nuclei and cytosol of the leaf epidermal and stomatal cells in all the lines under LL conditions (Fig. 10C). Moreover, growth under HL had no significant effect on the degree of oxidation (or the glutathione redox potentials; data not shown) of the nuclei and cytosol of the leaf epidermal or stomatal cells in any of the lines (Fig. 10D).

## Discussion

In previous studies, we have reported that the low ascorbate mutants *vtc-1* and *vtc2-1* are smaller than the wild type under short photoperiod growth conditions (Pastori *et al.*, 2003; Kerchev *et al.*, 2011) and we have shown that the lower rosette biomass accumulation was related to a smaller cell size in the mature leaves (Pavet *et al.*, 2005). However, the validity of using single mutations in each gene was recently questioned, and the decreased growth phenotype observed in *vtc2-1* was suggested to be due to an independent cryptic mutation and is not due to ascorbate deficiency (Lim *et al.*, 2016). In order to reconcile the issue of the role of ascorbate in the regulation of leaf size and biomass accumulation, we compared the shoot phenotypes of two ascorbate synthesis mutants (*vtc2-1* and *vtc2-4*) and the wild type under short-day growth conditions. Both low ascorbate mutants had a significantly smaller leaf size and rosette biomass accumulation than the wild type under the short-day growth conditions used in these studies (Figs 1, 2). However, this phenotype is clearly dependent on day length because the phenotype is less marked when plants are grown under LDs or constant light conditions (de Simone *et al.*, 2015; Lim *et al.*, 2016). Moreover, the suggestion that independent cryptic mutations in *vtc2-1* may be responsible for some of the traits that had previously been reported, we focused our efforts on a re-analysis of the role of ascorbate in photosynthesis by comparing pigment composition, NPQ, and photodamage in the leaves of *vtc2-1* and *vtc2-4* mutants relative to those of the wild type in plants grown under LL and HL conditions.

Photosynthesis and associated metabolism adjust rapidly to changes in light intensity (Cohu *et al.*, 2014; Demmig-Adams *et al.*, 2014). Following transitions from darkness or LL to HL, a period of photosynthetic activation is required that involves imbalances in both energy and redox status (Foyer *et al.*, 1992, 2017, Foyer and Noctor, 2000, 2009). It had long been considered that the photosynthetic apparatus can respond quickly to changes in irradiance in order to harvest light efficiently under LL while avoiding photo-damage under HL. However, recent evidence suggests that this is not the case and that leaves adjust photosynthetic efficiency in response to changing irradiance relatively slowly. Hence, decreasing the time required for NPQ relaxation was shown to increase the efficiency of CO_2_ assimilation in tobacco leaves to such an extent that productivity was increased by up to 20% (Kromdijk *et al.*, 2016). Moreover, wheat leaves were shown to take 15 min to regain maximum photosynthetic efficiency following transfer from shade to sun conditions, a major limitation being the time required to activate Rubisco (Taylor and Long, 2017). The adjustments required to accommodate transitions to HL were predicted to limit the productivity of the wheat crop by ~21% (Taylor and Long, 2017). We have recently shown that pre-illumination of the tomato shoot apex alone can accelerate photosynthetic induction in distal leaves (Guo *et al.*, 2016). This systemic induction of photosynthesis involves a phytochrome B-mediated auxin pathway that leads to H_2_O_2_ production in the systemic leaves accompanied by increased cyclic electron flow around PSI, allowing better adaptation to a changing light environment (Guo *et al.*, 2016). Similarly, altering the apoplastic ascorbate/DHA ratios by manipulating ascorbate oxidase activities was sufficient to alter the ability of photosynthesis to acclimate to high light (Karpinska *et al.*, 2017*a*). Tobacco leaves with low ascorbate oxidase activities and hence more ascorbate in the apoplast were able to maintain higher photosynthesis rates under HL than wild-type plants or plants with high ascorbate oxidase activities (Karpinska *et al.*, 2017*a*). Taken together, such findings demonstrate that light acclimation is responsive to local and systemic cues, as well as processes directly in the chloroplasts exposed to changing irradiance. The data presented here show that although Arabidopsis leaves under HL accumulate much higher levels of ascorbate than under LL, the process of acclimation to HL in the thylakoid membrane does not require high levels of leaf ascorbate. Moreover, low ascorbate does not lead to increased oxidation of the cytosol or nuclei, as measured in vivo using a redox-sensitive GFP probe, even under HL conditions. These observations show that ascorbate deficiency alone does not lead to chronic oxidation of leaf cells. The leaves of the *vtc2-1* and *vtc2-4* mutants had significantly lower levels of leaf ascorbate than those of the wild type under LL and HL conditions (Fig. 5). We have previously shown that like the wild type, the levels of leaf ascorbate increase significantly when these mutants are grown under continuous light (de Simone *et al.*, 2015). However, the leaves of both mutant genotypes still have only ~50% of the wild-type ascorbate even under continuous light growth conditions (de Simone *et al.*, 2015).

Under HL, leaves require less chlorophyll to maintain high photosynthesis rates than under LL, but such adjustments are very slow, taking several days (Yin and Johnson 2000; Karpinska *et al.*, 2017*a*). The leaves of the wild-type Arabidopsis plants in the present study had significantly lower levels of chlorophyll but much higher ascorbate contents after 7 d growth under HL compared with those grown under the LL regime. The increase in leaf ascorbate levels under high light is consistent with literature evidence showing the effect of light on ascorbate synthesis and accumulation (Bartoli *et al.*, 2006, 2009). Crucially, the data presented here demonstrate that the HL-dependent increases in leaf ascorbate were absent from the *vtc2-1* and *vtc2-4* mutants, illustrating the importance of GDP-l-galactose phosphorylase in light regulation of the ascorbate synthesis pathway. We have recently shown that tobacco leaves retained very high levels of ascorbate over the whole of the first photoperiod followinbg transfer to a LL environment after a period of growth under HL (Karpinska *et al.*, 2017*a*). Hence, ascorbate synthesis and accumulation are slow to acclimate to a changing light environment, even though the levels of transcripts encoding biosynthetic enzymes were significantly lower following the transition from HL to LL (Karpinska *et al.*, 2017*a*). The data presented here for the wild-type plants, which retained much higher levels of ascorbate in their leaves 2 d after the transition from HL to LL growth conditions (Fig. 5), support this conclusion.

The data presented here show that the low levels of leaf ascorbate in the *vtc2-1* and *vtc2-4* leaves led to a significant decrease in leaf anthocyanin contents. This finding is consistent with literature evidence showing that ascorbate is an important regulator of anthocyanin synthesis (Bashandy *et al.*, 2009; Page *et al.*, 2012). Ascorbate regulates the expression of genes involved in flavonol and anthocyanin precursor synthesis (Vanderauwera *et al.*, 2005; Page *et al.*, 2012) such as PHENYLALANINE AMMONIA-LYASE1 (PAL1), 4-COUMARATE:COENZYME A LIGASE3, CHALCONE SYNTHASE (CHS), as well as the MYB transcription factor PAP1 and an ELONGATED HYPOCOTYL5 (HY5) homologue HYH (Munné-Bosch *et al.*, 2013). The anthocyanin synthesis pathway was also suppressed in double mutants lacking the two plastid 2-Cys PRXs (*2cpa 2cpb*; Munné-Bosch *et al.*, 2013). However, the impaired capacity to accumulate anthocyanin observed in the leaves of the *vtc2-1* and *vtc2-4* mutants had no detectable effect on the effectiveness of the photo-protective processes measured within the leaves under LL conditions (Fig. 7).

Previous studies concerning the effects of low ascorbate on photosynthesis using *vtc2-1* mutants (e.g. Müller-Moulé *et al.*, 2003) have reported that a sudden exposure to high irradiance caused photoinhibition and photooxidation compared with the wild type. Moreover, long-term acclimation of *vtc2-1* to HL was accompanied by a noticeable inhibition of photosynthesis (Müller-Moulé *et al.*, 2003). The data presented here confirm these observations but reveal some important differences in the responses of *vtc2-1* and *vtc2-4* mutants to HL (Figs 4, 8). In particular, the *vtc2-1* had significantly lower qPD values than the *vtc2-4* mutants under HL. Moreover, the HL-dependent changes in leaf pigment content and composition were different in the *vtc2-1* and *vtc2-4* leaves, even though both mutants have the same low level of ascorbate relative to the wild type. The HL-dependent changes in qPD values and in leaf pigment content and composition were similar in the *vtc2-4* and wild-type leaves. This finding suggests that the *vtc2-1* mutant has enhanced susceptibility of photosynthesis to photoinhibition and photooxidation as previously reported (Müller-Moulé *et al.*, 2003, 2014), but it is not caused by the low ascorbate content of the leaves. While the *vtc2-1* leaves showed significantly lower qP_d_ values under HL, indicative of a greater amount of damage, there was no consistent effect of low ascorbate on this parameter in the *vtc2-1* and *vtc2-4* mutants.

The leaves of the *vtc2-4* mutants accumulated significantly more zeaxanthin than the wild type under HL (Fig. 10). This finding suggests that ascorbate is not the only reductant that can drive the vioxanthin de-epoxidase reaction under HL. Vioxanthin de-epoxidase has a low affinity for ascorbate and this is strongly pH dependent, the enzyme being saturated only at 10–20 mM ascorbate at pH 4.5–5.5 (Bratt *et al.*, 1995; Jahns *et al.*, 2009). Moreover, the enzyme requires much higher levels of ascorbate for saturation (100 mM) at pH 6.0 (Bratt *et al.*, 1995; Jahns *et al.*, 2009). While chloroplasts have ~10 mM ascorbate, the levels in the thylakoid lumen are much lower (Zechmann *et al.*, 2011; Heyneke *et al.*, 2013). Hence, vioxanthin de-epoxidase activity must always be limited by ascorbate availability in the wild type even under optimal conditions. Taken together these observations suggest that alternative reductants are available to drive the vioxanthin de-epoxidase reaction. Arabidopsis mutants lacking both the stromal (sAPX) and thylakoid (tAPX) forms of APXs show a similar sensitivity to HL stress to the wild type (Giacomelli *et al.*, 2007; Kangasjärvi *et al.*, 2008; Maruta *et al.*, 2010). These APXs work together with PRXs to remove hydrogen peroxide produced by the photosynthetic electron transport chain (Awad *et al.*, 2015). Mutants lacking the thylakoid 2-Cys PRXs (*2cpa 2cpb*) and a triple mutant deficient in 2-Cys PRXs and thylakoid APX (*2cpa 2cpb tapx*) showed much lower photosynthetic efficiencies than the wild type under HL conditions (Awad *et al.*, 2015). As in the case of hydrogen peroxide removal, multiple reductants might support the vioxanthin de-epoxidase reaction and hence zeaxanthin formation. Moreover, low ascorbate had no marked effect on the degree of cellular oxidation (Fig. 8), confirming that other antioxidants are able to compensate for low ascorbate in the maintenance of cellular redox homeostasis as suggested previously (Müller-Moulé *et al.*, 2014). However, ascorbate deficits influence photosynthetic gene expression (Kiddle *et al.*, 2003) even though glutathione levels are increased to compensate for low ascorbate and maintain antioxidant capacity (Pavet *et al.*, 2005).

The finding that the degree of oxidation in the nuclei and cytosol in the leaf epidermal and stomatal cells measured using roGFP (Meyer *et al.*, 2007) was similar in the low ascorbate lines and the wild type both at LL and at HL is consistent with the conclusion that other antioxidants are increased to compensate for low ascorbate (Pavet *et al.*, 2005). We have not used the next-generation glutaredoxin (GRX)-containing roGFP2 biosensor probes that were developed for the measurement of the glutathione redox potential in severely glutathione-deficient mutants (Aller *et al.*, 2013). Hence, the effectiveness of the probe is determined by the local levels of GRX proteins. Like the cytosol, the nucleus has a number of GRX forms, including GRXC7, GRXC8, and GRXC9, which are involved in different processes such as pathogen responses and petal development through interactions with TGA transcription factors (Rouhier, 2010). Moreover, it is likely that at least some if not all of these GRX forms, such as GrxC7 (also called ROXY1), have a dual nucleo-cytoplasmic localization and partition between the two compartments. We were unable to undertake accurate roGFP measurements in leaf mesophyll cells because of interference from autofluorescence of the photosynthetic pigments, even though the roGFP2 emission peak is well separated from the chlorophyll emission peak. While the oxidation status of the cytosol and nuclei of the epidermal cells cannot be used to assess photodamage in the mesophyll cells, we have previously shown that inhibitors of chloroplast functions underpinning the efficient operation of photosynthesis, such as norflurazon (NF) and lincomycin (LINC), cause marked increases in the degree of oxidation of the nuclei and cytosol of the epidermal and stomatal guard cells (Karpinska *et al.*, 2017*b*). The degree of oxidation in the nuclei and cytosol of the leaf epidermal and stomatal cells was decreased to 50% in the presence of both LINC and NF compared with less than ~10% in untreated controls (Karpinska *et al.*, 2017*b*). The levels of glutathione have previously been shown to be increased in low ascorbate mutants (Pavet *et al.*, 2005). Thus, the present finding that the degree of oxidation was similar in the nuclei and cytosol of the leaf epidermal and stomatal guard cells of all lines is consistent with previous observations indicating that cells with low ascorbate are protected from increased oxidation by increases in the levels of other antioxidants.

The data presented here not only confirm the role of ascorbate in the regulation of rosette growth but they also illustrate the importance of analysing more than one mutant in any single gene to verify relationships between traits and gene functions. While the data regarding the responses of the *vtc2-1* mutants to HL are similar to those reported previously (Müller-Moulé *et al.*, 2003, 2014), the lack of corroboration in the *vtc2-4* mutant suggests that low ascorbate is not the basis for the enhanced sensitivity to photoinhibition and photooxidation reported in the *vtc2-1* mutants. Moreover, the data presented here suggest that overall the ascorbate mutants are less susceptible to HL-induced photodamge than the wild type, a finding which again may illustrate the importance of functional redundancy in systems that protect the photosynthetic apparatus.

## Abbreviations:

APX: ascorbate peroxidase
FR: far red light
HL: high light
LL: low light
NPQ: non-photochemical quenching of chlorophyll *a* fluorescence
PAM: pulse amplitude modulation
PRX: peroxiredoxin
qP_d_: photochemical quenching in the dark
RCII: PSII reaction centre
roGFP: redox-sensitive green fluorescent protein
SP: saturating pulse.
